# Supplementary material for: MxB is an interferon-induced restriction factor of human herpesviruses
Source: Nat Commun. 2018 May 17;9:1980. doi: 10.1038/s41467-018-04379-2 (PMC5958057; doi:10.1038/s41467-018-04379-2)
Supplement: Supplementary file 1 — Supplementary Information [file 41467_2018_4379_MOESM1_ESM.pdf]

Supplementary Information

MxB is an interferon-induced restriction factor of human herpesviruses

Crameri M. *et al.*

Supplementary Methods

Cell lines, plasmids, viruses, siRNAs, and antibodies. Primary human umbilical vein endothelial cells (HUVEC, CRL-1730, ATCC, kindly provided by Mirco Ponzoni, IRCCS Istituto G. Gaslini, Genova, Italy) were cultured on plates pre-coated with collagen derived from rat tail tendon (Merck) in HUVEC medium (EBM-2 Endothelial Growth Basal Medium (CC-3156, Lonza) supplemented with EGM-2 Endothelial Growth SingleQuot Kit Supplement & Growth Factors (CC-4176, Lonza)) at 37 °C and 5% CO_2. The puromycin resistance plasmid pCDNA3.1-Puro(+) was generated from pCDNA3.1-Neo(+) by replacing the neomycin resistance cassette with a puromycin resistance cassette. The constitutive luciferase reporter plasmid pIS1-Eef25UTR-renilla was kindly provided by Ben Hale. The HIV-1-based VSV-G-pseudotyped single-cycle luciferase reporter virus NL-Luc was generated by transfecting HEK-293T cells with pNLluc-AM vector[1] and pVSV-G and titres were determined by ELISA. HAdV-C5 (wt300) and HAdV-C5_dE1_GFP carrying a GFP cassette under the control of the cytomegalovirus major immediate-early promoter in place of the E1 region have previously been described[2]. The siRNAs directed against dsRNA-dependent protein kinase R (*PKR*) and interferon regulatory factor 9 (*IRF9*) were purchased from Qiagen (siPKR #1, Cat. No. SI02223011, target sequence 5'-ACG GAA AGA CTT ACG TTA TTA-3'; siPKR #2, Cat. No. SI02223018, target sequence 5'-CGG AAA GAC TTA CGT TAT TAA-3'; siIRF9, Cat. No. SI00084371, target sequence 5'-CAA CAA GAG TTC TGA ATT TAA-3'). Primary antibodies used in addition to those stated in the main article or used in alternative assays were rabbit anti-PKR (Abcam, ab32052, 1:1,000), rabbit anti-HAdV protein VI PAb[3], rabbit anti-HSV-1 VP22 PAb AGV031[4] (kindly donated by G. Elliott, University of Surrey, Guildford, United Kingdom, 1:2,500), mouse anti-α-Tubulin (Sigma-Aldrich, T6074, 1:5,000), rabbit anti-Histone H3 (Abcam, ab1791, 1:2,500), rabbit anti-HC PAb (directed against DNA-containing HSV-1 capsids, kindly donated by R. Eisenberg and G. Cohen, University of Pennsylvania, Philadelphia, USA, 1:1,000 in immunofluorescence assay and immunoblot assay), and mouse anti-HSV-1/2 VP5 (Abcam, ab6508, 1:3,000 in immunoblot assay). Densitometric analyses were performed using MultiGauge version 3.0. Values were normalized to the loading control and are represented relative to the appropriate control condition. Uncropped scans of immunoblots can be found in Supplementary Figs. 9-12.

KSHV infection of HUVECs. HUVECs were seeded on cell culture plates pre-coated with collagen derived from rat tail tendon (Merck). Cells were allowed to settle for 24 h and were then transfected with siIRF9 (to prevent unspecific activation of ISGs) together with siNT or siMxB #2 in equal shares in HUVEC medium. Transfection complexes were prepared using a total of 10 nM siRNA and 2 µl Lipofectamine RNAiMAX reagent (Life Technologies) in a 12-well format. At 48 h post transfection, cells were transduced with GST or MxB lentiviruses in OptiMem reduced serum medium (Life Technologies) and HUVEC medium in equal shares together with 8 µg/ml polybrene (Sigma-Aldrich). At 48 h post transduction, cells were infected with KSHV (MOI=0.5) in EBM-2 Endothelial Growth Basal Medium (CC-3156, Lonza) supplemented with 10% FBS and 8 µg/ml polybrene (Sigma-Aldrich). Cells were spinoculated at 800×*g* and 4 °C for 30 min and then shifted to 37 °C and 5% CO_2. 24 h post infection, cells were fixed with 1% (w/v) PFA and GFP expression was measured on a LSR II Fortessa flow cytometer (BD Biosciences) and analysed using FlowJo version 10.2.

Subcellular fractionation and HSV-1 genomic qPCR. A549-MxB cells were siRNA-transfected and then inoculated with HSV-1 in infection medium for 60 min on ice in presence of 100 µg/ml cycloheximide (Sigma-Aldrich). Cells were washed once in PBS and incubated in infection medium with 100 µg/ml cycloheximide for 4 h. After two washing steps in ice-cold PBS, cytoplasmic and nuclear fractions were prepared as follows. Cells were incubated with sucrose lysis buffer (10 mM HEPES pH=7.9, 340 mM sucrose, 3 mM $CaCl_2$, 2 mM magnesium acetate, 0.1 mM EDTA, and cOmplete™ Protease Inhibitor Cocktail (Roche Applied Science)) containing 0.5% NP-40 for 10 min on ice. Samples were centrifuged at 3,500×*g* and 4 °C for 5 min. Supernatants were stored as cytoplasmic fractions. Remaining pellets were washed twice in sucrose buffer without NP-40. Extraction of cytoplasmic and nuclear DNA was performed with 50% of each supernatant and pellet fraction, respectively, using DNeasy Blood & Tissue kit (Qiagen) according to the manufacturer's instructions. The other 50% of each supernatant and pellet fraction were subjected to immunoblot assay. HSV-1 genomic DNA was quantified by qPCR using *GAPDH* gene as endogenous control and primers specific for the HSV-1 *US4* gene together with appropriate TaqMan® probes (Applied Biosystems). HSV-1 genomic DNA sequences were amplified at the following conditions: 2 min 50 °C, 10 min 95 °C, 50×(15 s 95 °C, 60 s 60 °C). The following primers and probes were used (in 5'-3' orientation): *US4*-forward: CTG TTC TCG TTC CTC ACT GCC T, *US4*-reverse: CAA AAA CGA TAA GGT GTG GAT GAC, *US4* probe: 6-FAM-CCG CCC TGG ACA CC-MGB NFQ; *GAPDH*-forward: CAA GGT CAT CCA TGA CAA CTT TG, *GAPDH*-reverse: GGC CAT CCA CAG TCT TCT GG, *GAPDH* probe: VIC-ACC ACA GTC CAT GCC ATC ACT GCC A-TAMRA. Ct values were normalized to *GAPDH* (ΔCt) and relative virus DNA levels were calculated using the $2^{-\Delta\Delta Ct}$ method.

NL-Luc infections. NL-Luc virus stock was diluted in OptiMem reduced serum medium (Life Technologies) together with 0.025% diethylaminoethyl (DEAE)-dextran (Sigma-Aldrich) for A549 cells or 8 µg/ml polybrene (Sigma-Aldrich) for HeLa cells. Cells were inoculated at 37 °C and 5% CO_2 for 90 min, then the inoculum was removed and replaced with growth medium, and the cells were incubated at 37 °C and 5% CO_2.

HAdV-C5 infections and readout. A549-GST and A549-MxB cells were infected with HAdV-C5 (wt300) or HAdV C5_dE1_GFP at an MOI of 0.4. At 20 h post infection, cells were fixed, stained with DAPI, and analysed for infection by microscopy according to previously published protocols[2,3,5].

Luciferase assays. For the HIV-1 reporter assay in HeLa cells, 75ng expression plasmids and 425 ng puromycin resistance plasmid were used. To prevent viral reporter expression in untransfected cells, 1 µg/ml puromycin (Invivogen) was added to the inoculum and to the post-infection medium. For the herpesvirus reporter assays related to phosphonoacetic acid (PAA, Sigma-Aldrich), time dependency, and MOI dependency, 200 ng pGL-T9G and 300 ng empty expression plasmid were used. After the infection period, luciferase activity was determined using the BrightGlo™ Luciferase Assay System (Promega) on an Envision 2104 plate reader (Perkin Elmer). For the constitutive reporter assay, 200 ng pIS1-Eef25UTR-renilla and increasing amounts of expression plasmids were used (37.5 ng, 75 ng, 150 ng). The total amount of DNA per well was adjusted using the empty expression plasmid. Luciferase activity was determined using the *Renilla*-Glo® Luciferase Assay System (Promega) on an Envision 2104 plate reader (Perkin Elmer).

Statistical analyses. Statistical analyses were performed as described in the main article using R version 3.4.1[6]. Correlation analysis was performed using the *cor* function in the *stats* package. For curve fitting, a generalized additive model was applied using the *qplot* function in the *ggplot2* package[7].

Supplementary References

1. Pugach, P. *et al.* HIV-1 clones resistant to a small molecule CCR5 inhibitor use the inhibitor-bound form of CCR5 for entry. *Virology* **361**, 212-228 (2007).
2. Suomalainen, M. *et al.* A direct and versatile assay measuring membrane penetration of adenovirus in single cells. *J Virol* **87**, 12367-12379 (2013).
3. Burckhardt, C. J. *et al.* Drifting motions of the adenovirus receptor CAR and immobile integrins initiate virus uncoating and membrane lytic protein exposure. *Cell host & microbe* **10**, 105-117 (2011).
4. Elliott, G. & O'Hare, P. Intercellular trafficking and protein delivery by a herpesvirus structural protein. *Cell* **88**, 223-233 (1997).
5. Stichling, N. *et al.* Lung macrophage scavenger receptor SR-A6 (MARCO) is an adenovirus type-specific virus entry receptor. *PLoS pathogens* **14**, e1006914 (2018).
6. R Core Team. R: A language and environment for statistical computing v. 3.4.1 (R Foundation for Statistical Computing, Vienna, Austria, 2017). http://www.R-project.org/.
7. Wickham, H. ggplot2: Elegant Graphics for Data Analysis (Springer-Verlag, New York, USA, 2009). http://ggplot2.org/.

Supplementary Fig. 1

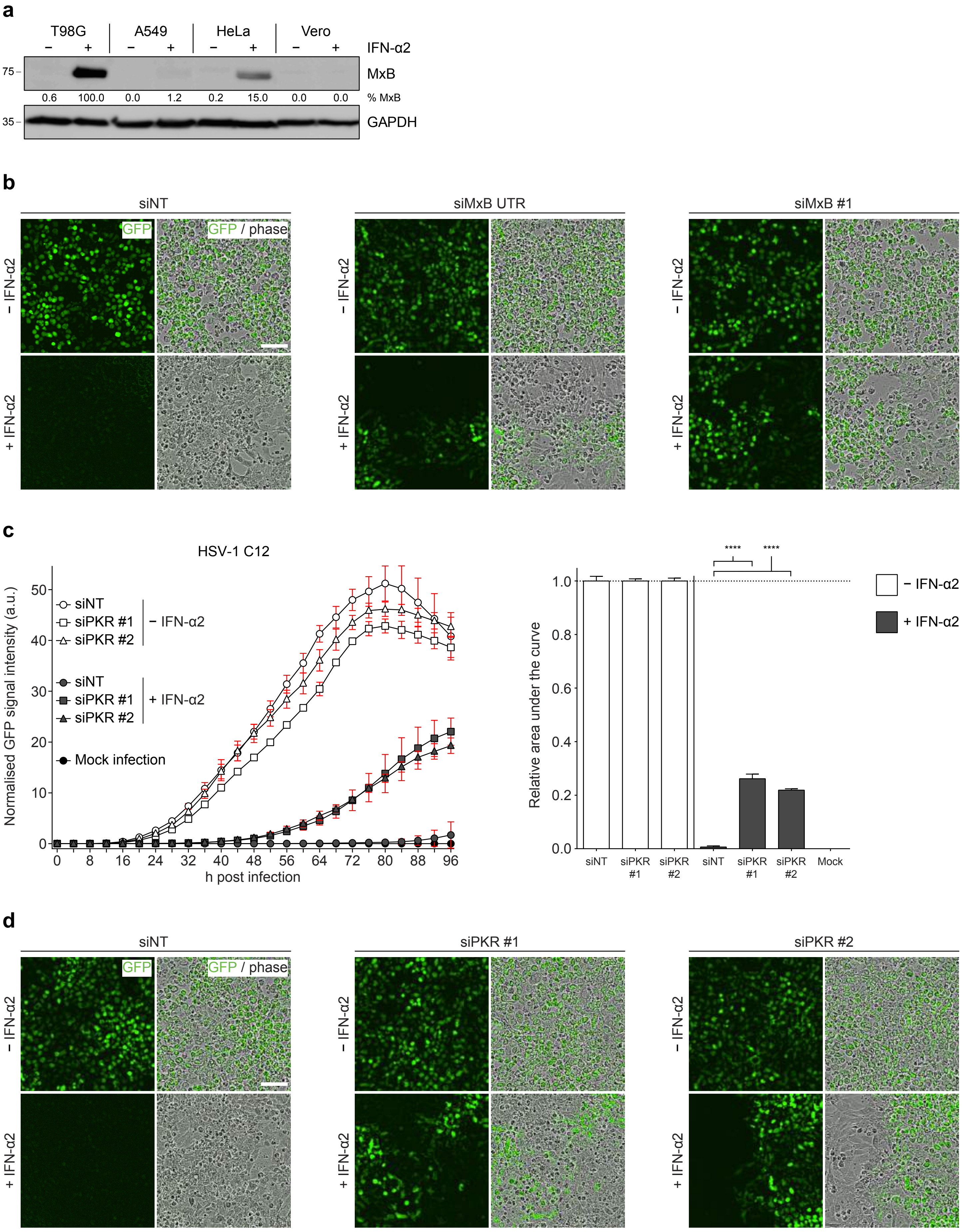

Supplementary Fig. 1 T98G cells are a valuable tool to study the effect of interferon-induced MxB and PKR on herpesvirus infection. **a** T98G, A549, HeLa, and Vero cells were mock-stimulated or stimulated with human IFN-α2 (1,000 IU ml^{-1}). At 18 h post stimulation, cells were lysed for immunoblot analysis of endogenous MxB protein expression. GAPDH served as loading control. **b** Refers to Fig. 1d: Representative micrographs of T98G cells pretreated with siRNA and human IFN-α2 and subsequently infected with HSV-1 strain C12 (MOI=0.1) at 56 h post infection. Scale bar, 100 µm. **c** T98G cells were transfected with non-targeting siRNA (siNT) or two different siRNAs targeting endogenous *PKR* (siPKR #1, siPKR #2). At 30 h post transfection, cells were mock-stimulated or stimulated with human IFN-α2 (500 IU ml^{-1}). At 48 h post transfection, cells were mock-infected or infected with HSV-1 recombinant strain C12 (MOI=0.1). Virus growth was determined by real-time quantification of GFP signal intensity. Left panel: Data points indicate the integrated green object intensity normalised to cell confluence (mean±s.d., n=3 biological replicates). Right panel: relative area under the curve from 0-96 h post infection. GFP signals of mock-stimulated samples are each set to 1. Bars indicate relative mean+s.d., n=3 biological replicates. ANOVA modelling relative area under the curve with IFN and siRNA as explanatory variables: IFN, $F(1,12)$=8,766.5, $p<0.0001$; siRNA, $F(2,12)$=230.5, $p<0.0001$; antagonising interaction, $F(2,12)$=230.5, $p<0.0001$. **d** Representative micrographs from **c** at 80 h post infection. Scale bar, 100 µm. All data are representative of two independent experiments. a.u., arbitrary units. Multiple comparisons: **** $p<0.0001$.

Supplementary Fig. 2

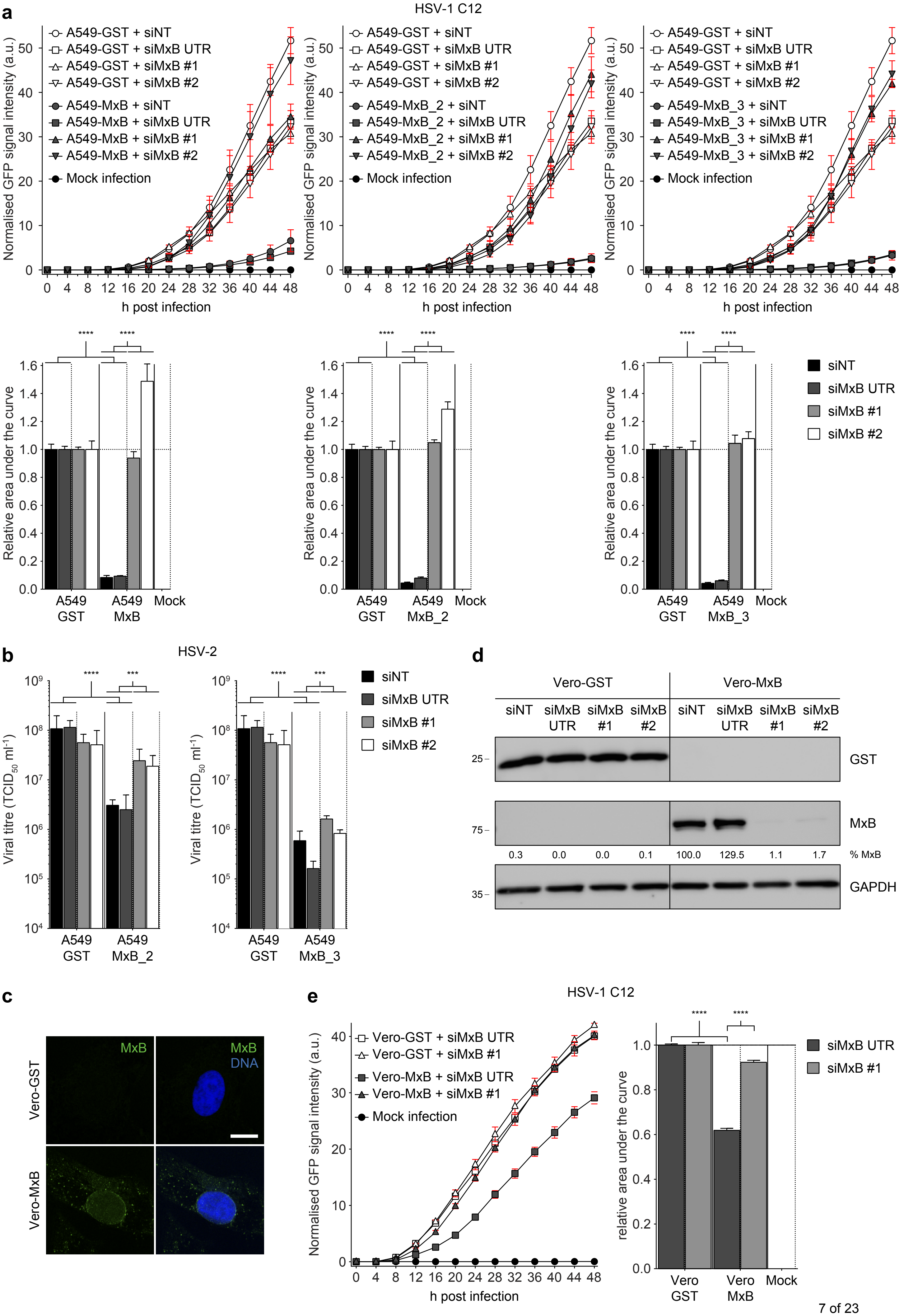

Supplementary Fig. 2 Ectopic expression of MxB inhibits HSV replication in three independent A549 clones and Vero cells. **a** A549-GST and A549-MxB cells plus two additional independent high-expression MxB clones (A549-MxB_2 & A549-MxB_3) were transfected with non-targeting siRNA (siNT), siRNA targeting the 3'-UTR of endogenous *MX2* (siMxB UTR), or two different siRNAs targeting the coding sequence of endogenous and overexpressed *MX2* (siMxB #1, siMxB #2). At 48 h post transfection, cells were mock-infected or infected with HSV-1 recombinant strain C12 expressing GFP (MOI=0.1). Virus growth was determined by real-time quantification of GFP signal intensity. Upper panels: Data points indicate the integrated green object signal intensity normalised to cell confluence (mean±s.d., n=3 biological replicates). Lower panels: relative area under the curve from 0-48 h post infection. GFP signals of infected A549-GST cells are each set to 1. Bars indicate relative mean+s.d., n=3 biological replicates. ANOVAs modelling square-rooted area under the curve with cell line and siRNA as explanatory variables: A549-GST vs. A549-MxB: cell line, $F(1,16)=853.3$, $p<0.0001$; siRNA, $F(3,16)=511.5$, $p<0.0001$, antagonising interaction, $F(3,16)=511.9$, $p<0.0001$. A549-GST vs. A549-MxB_2: cell line, $F(1,16)=2,176$, $p<0.0001$; siRNA, $F(3,16)=1,125$, $p<0.0001$, antagonising interaction, $F(3,16)=1,126$, $p<0.0001$. A549-GST vs. A549-MxB_3: cell line, $F(1,16)=2,028.2$, $p<0.0001$; siRNA, $F(3,16)=790.6$, $p<0.0001$, antagonising interaction, $F(3,16)=790.9$, $p<0.0001$. **b** A549-GST, A549-MxB_2 and A549-MxB_3 cells were siRNA-transfected as in **a** but then infected with HSV-2 strain G (MOI=0.05) for 48 h. Viral output was measured by $TCID_{50}$ assay. Data are representative of two independent experiments. Bars indicate mean+s.d., n=3 biological replicates. ANOVAs modelling log-transformed titres with cell line and siRNA as explanatory variables: A549-GST vs. A549-MxB_2: cell line, $F(1,16)=391.189$, $p<0.0001$; siRNA, $F(3,16)=2.023$, $p=0.15124$; antagonising interaction, $F(3,16)=8.77$, $p=0.00114$. A549-GST vs. A549-MxB_3: cell line, $F(1,16)=58.122$, $p<0.0001$; siRNA, $F(3,16)=1.481$, $p=0.2573$; antagonising interaction, $F(3,16)=7.54$, $p=0.0023$. Multiple comparisons: *** $p<0.001$; **** $p<0.0001$. **c** Vero cells were engineered to stably express GST or human MxB. Vero-GST and Vero-MxB cell lines were generated from clones with high gene expression. MxB protein expression and intracellular localisation was assessed by immunostaining. Nuclei were stained with Hoechst 33342. Scale bar, 10 µm. **d** Vero-GST and Vero-MxB cells were transfected with the indicated siRNAs. At 48 h post transfection, cells were lysed for immunoblot analysis of GST and MxB protein expression and *MX2* silencing efficiency. GAPDH served as loading control. **e** Vero-GST and Vero-MxB cells were transfected with the indicated siRNAs. At 48 h post transfection, cells were infected with recombinant HSV-1 strain C12 (MOI=0.5). Virus growth was determined by real-time quantification of GFP signal intensity. Data are representative of two independent experiments. Left panel: Data points indicate the integrated green object signal intensity normalised to cell confluence (mean ±s.d., n=3 biological replicates). Right panel: relative area under the curve from 0-48 h post infection. GFP signals of infected Vero-GST cells are each set to 1. Bars indicate relative mean +s.d., n=3 biological replicates. Unpaired two-tailed Student's t-tests: **** $p<0.0001$. a.u., arbitrary units.

Supplementary Fig. 3

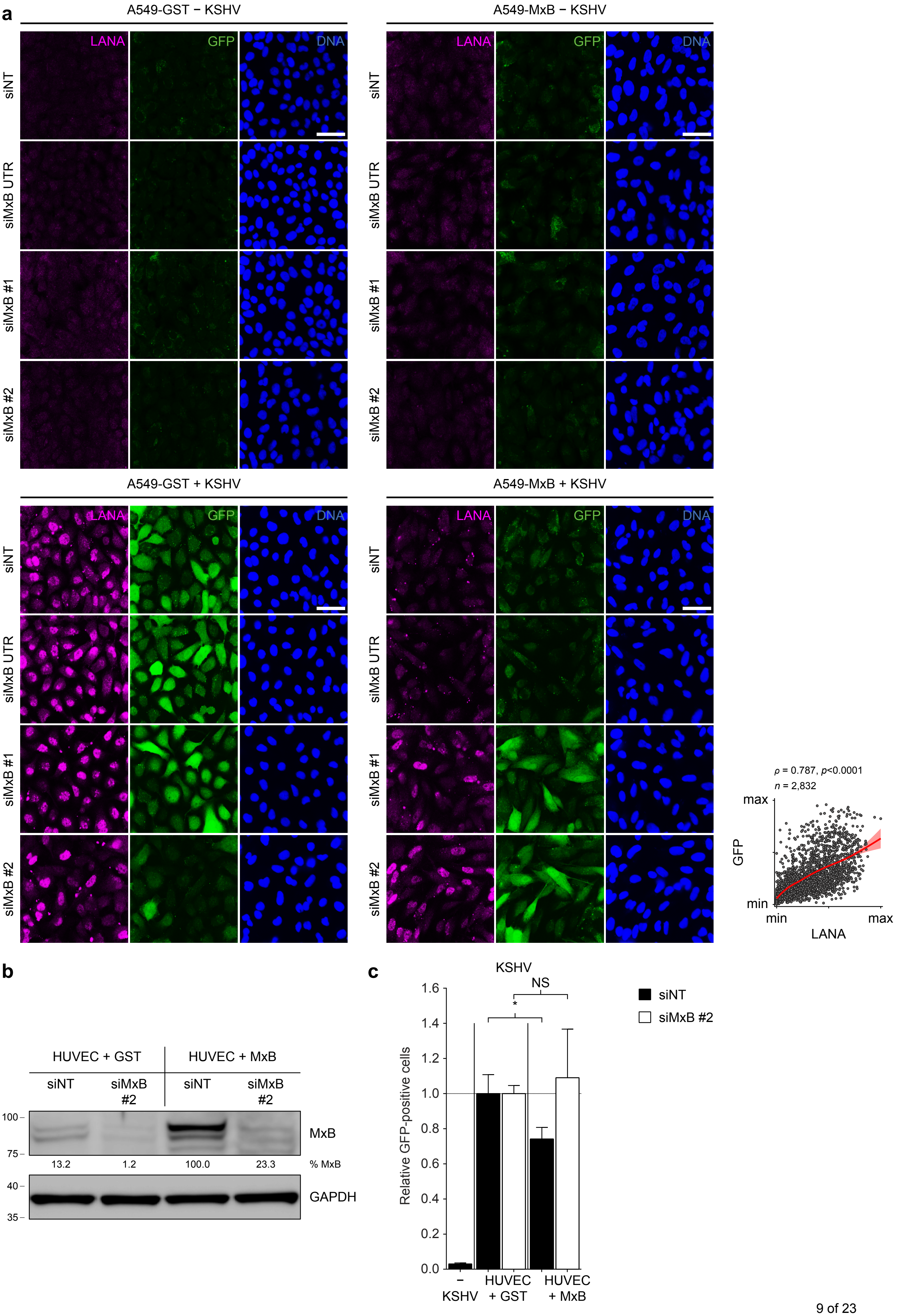

Supplementary Fig. 3 Ectopic expression of MxB inhibits KSHV infection in A549 cells and in human umbilical vein endothelial cells. **a** Refers to Fig. 3b: Representative micrographs of A549-GST and A549-MxB cells pretreated with siRNA and subsequently mock-infected or infected with recombinant KSHV expressing GFP (MOI=0.5) at 16 h post infection. Endogenous LANA protein expression is shown in magenta and GFP reporter expression is shown in green. Nuclei were stained with Hoechst 33342. Scale bar, 40 µm. Bottom right panel: Intranuclear LANA signal intensity was plotted against the corresponding GFP signal intensity for each cell and Spearman rank correlation test was performed. The red smoother line describes LANA signal intensity as a function of GFP signal intensity along with the 95% confidence intervals (red shading) as predicted by a generalised additive model. ρ Spearman's rho. **b** Human umbilical vein endothelial cells (HUVECs) were transfected with siRNA targeting *IRF9* together with non-targeting siRNA (siNT) or siRNA targeting the coding sequence of endogenous and overexpressed *MX2* (siMxB #2). At 48 h post transfection, cells were transduced with lentiviruses encoding GST or MxB. At 48 h post transduction, cells were lysed for immunoblot analysis of MxB protein expression and *MX2* silencing efficiency. GAPDH served as loading control. **c** HUVECs were transfected and transduced as in **b**. At 48 h post transduction, cells were infected with recombinant KSHV expressing GFP (MOI=0.5) for 24 h. Viral output was measured by flow cytometry. Data are representative of two independent experiments. The fractions of GFP-positive cells in GST-transduced samples are each set to 1. Bars indicate relative mean+s.d., n=3 biological replicates. Unpaired two-tailed Student's t-tests: NS $p \geq 0.05$; * $p < 0.05$.

Supplementary Fig. 4

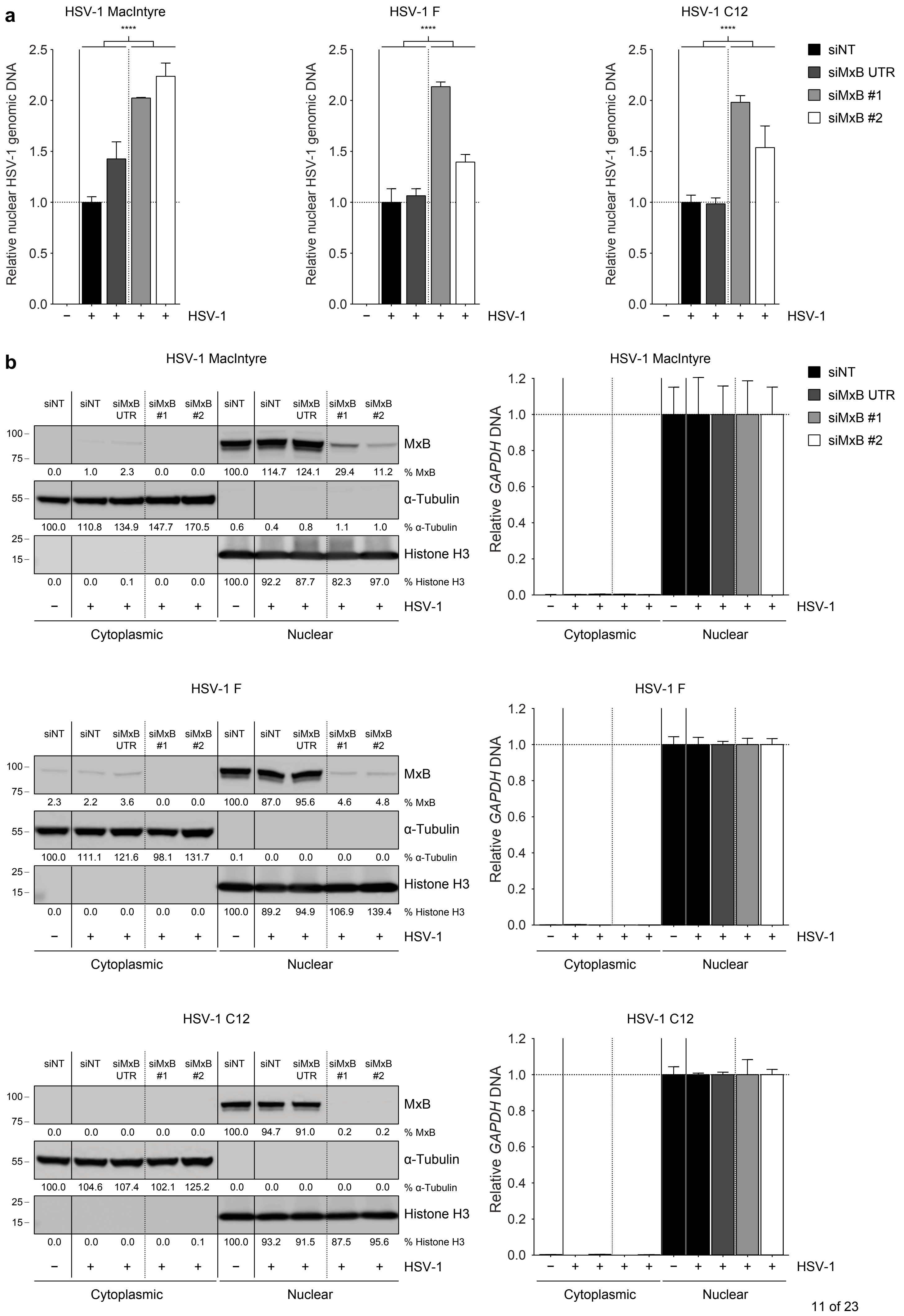

Supplementary Fig. 4 Silencing of *MX2* enhances nuclear uptake of HSV-1 genomic DNA during virus entry. **a** A549-MxB cells were transfected with the indicated siRNAs. At 60 h post transfection, cells were mock-infected or infected with HSV-1 strain MacIntyre (left panel), F (middle panel), or C12 (right panel) (MOI=0.5) for 4 h in presence of cycloheximide. Cytoplasmic and nuclear preparations were prepared and DNA was extracted from nuclear preparations for RT-qPCR analysis of HSV-1 genomic sequences. Data are representative of one (MacIntyre) or three (F & C12) independent experiments. Bars indicate mean+s.d., n=3 technical replicates. Values from siNT-transfected, infected samples are each set to 1. One-way ANOVAs modelling siRNA as explanatory variable: HSV-1 MacIntyre, $F(3,8)=80.28$, $p<0.0001$; HSV-1 F, $F(3,8)=109.2$, $p<0.0001$; HSV-1 C12, $F(3,8)=48.04$, $p<0.0001$. Multiple comparisons: **** $p<0.0001$. **b** Left panels: Cytoplasmic and nuclear preparations from **a** were subjected to immunoblot analysis to control for MxB protein expression, *MX2* silencing efficiency, and fractionation efficiency. A-Tubulin served as cytoplasmic marker and Histone H3 served as nuclear marker. Right panels: Cytoplasmic and nuclear preparations from **a** were subjected to DNA extraction and RT-qPCR of genomic *GAPDH*, revealing no leakage of genomic material from the nucleus during sample preparation. Bars indicate mean+s.e.m., n=3 technical replicates. For each group, values from nuclear fractions are set to 1.

Supplementary Fig. 5

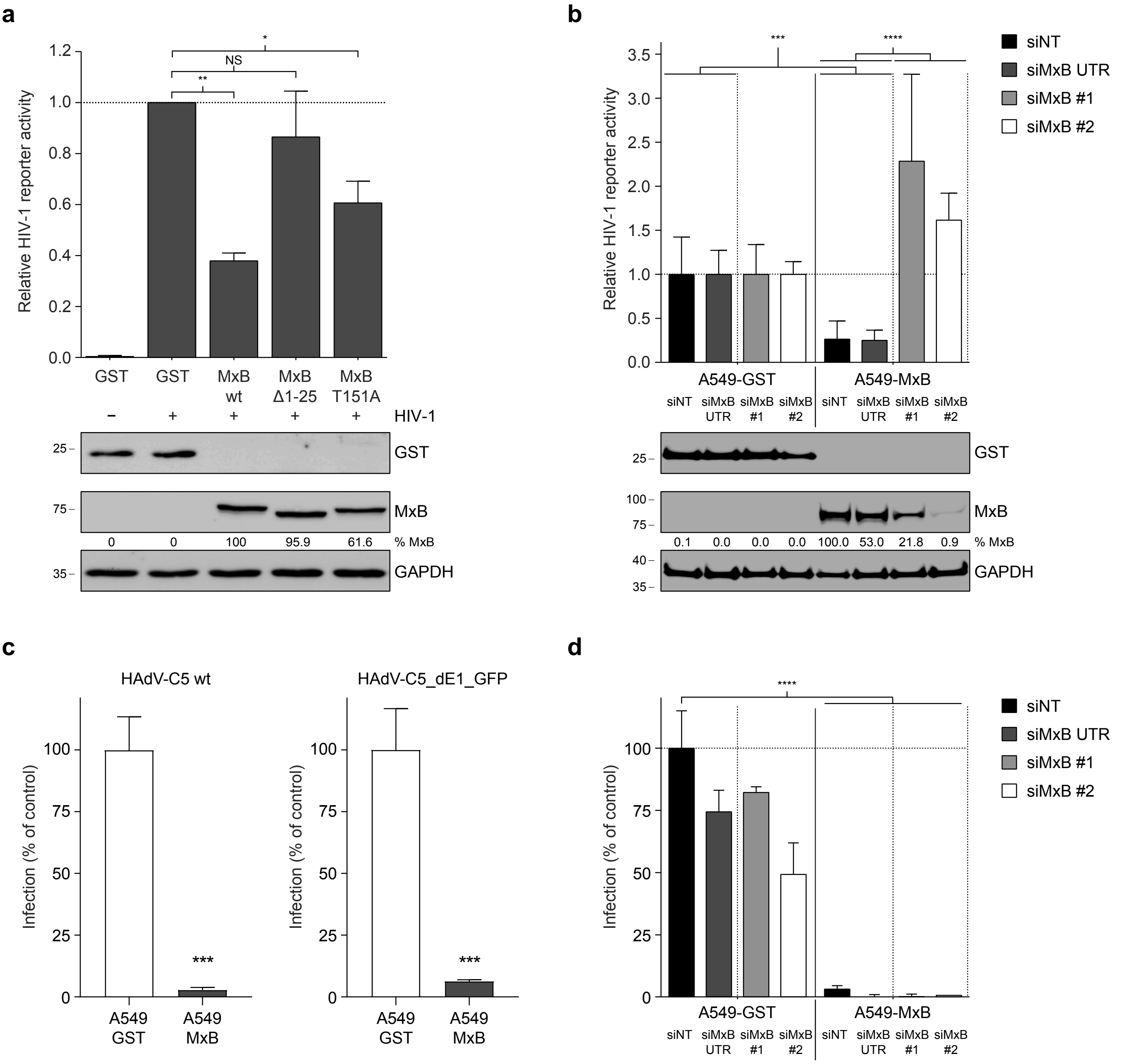

Supplementary Fig. 5 Effect of ectopic MxB expression on HIV-1 pseudovirus and HAdV-C5 single-round infection. **a** HeLa cells were transiently transfected with expression plasmids encoding the indicated proteins. At 24 h post transfection, cells were mock-infected or infected with HIV-1-based luciferase reporter virus NL-Luc (50 fg p24 equivalents per cell). Virus infection efficiency was determined by luciferase assay at 48 h post infection and is represented relative to the GST control. Bars indicate mean+s.e.m., *n*=3 independent experiments with three biological replicates each. Unpaired two-tailed Welch's *t*-tests: NS $p \geq 0.05$; * $p<0.05$; ** $p<0.01$. GST and MxB protein expression was controlled by immunoblot analysis using pooled samples for each condition. GAPDH served as loading control. **b** A549-GST and A549-MxB cells were infected with HIV-1-based luciferase reporter virus NL-Luc (100 fg p24 equivalents per cell). Virus infection efficiency was determined by luciferase assay at 48 h post infection. For each group, values from A549-MxB cells are represented relative to values from A549-GST cells. Bars indicate mean +s.d., *n*=3 biological replicates. ANOVA modelling square-rooted relative reporter activity with cell line and siRNA as explanatory variables: cell line, $F(1,16)=0.604$, $p=0.448514$; siRNA, $F(3,16)=11.566$, $p=0.000279$; antagonising interaction, $F(3,16)=11.37$, $p=0.000305$. Multiple comparisons: *** $p<0.001$; **** $p<0.0001$. GST and MxB protein expression as well as *MX2* silencing efficiency was controlled by immunoblot analysis using pooled samples for each condition. GAPDH served as loading control. **c** A549-GST and A549-MxB cells were infected with HAdV-C5 wt or HAdV-C5 carrying a GFP cassette under the control of the cytomegalovirus major immediate-early promoter in place of the E1 region (HAdV-C5_dE1_GFP) at an MOI of 0.4. At 20 h post infection, cells were fixed, stained with DAPI, and analysed for infection by microscopy. For HAdV-C5 wt, cells were stained against HAdV protein VI, while for HAdV-C5_dE1_GFP, GFP expression was used as a marker for infection. For each group, values from A549-MxB cells are represented in % relative to values from A549-GST cells. Unpaired two-tailed Student's *t*-tests: *** $p<0.001$. **d** A549-GST and A549-MxB cells were reverse-transfected with the indicated siRNAs for 48 h and subsequently infected with HAdV-C5-GFP for 20 h. Infection analysis was performed as in **c**. One-way ANOVA modelling relative infection levels with Holm-Sidak's multiple comparisons test. Bars indicate mean+s.d., *n*=3 biological replicates. *** $p<0.001$; **** $p<0.0001$.

Supplementary Fig. 6

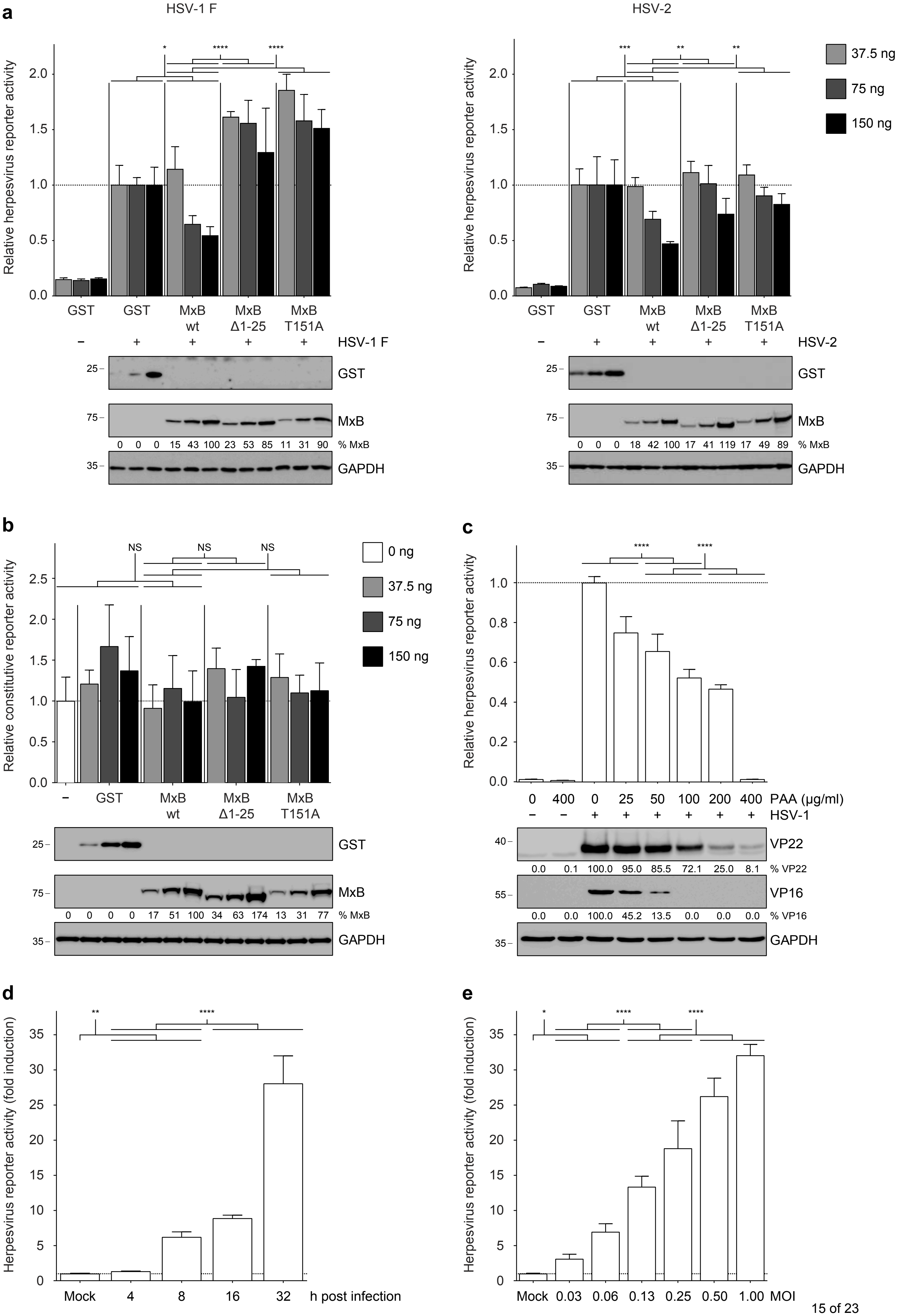

Supplementary Fig. 6 Transient transfection of the herpesvirus-specific luciferase reporter plasmid pGL-T9G facilitates the study of lytic replication kinetics under various conditions. **a** HeLa cells were transiently transfected with pGL-T9G reporter plasmid together with increasing amounts of expression plasmids encoding the indicated proteins. At 24 h post transfection, cells were mock-infected or infected with HSV-1 strain F or HSV-2 strain G (MOI=0.5). Virus infection efficiency was determined by luciferase assay at 32 h post infection and is presented relative to the respective conditions with GST expression. Luciferase activity from all conditions is normalised to mock-infected cells. Data related to HSV-1 F are representative of three independent experiments. Bars indicate mean+s.d., $n=3$ biological replicates. ANOVAs modelling relative reporter activity with protein variant and dose as explanatory variables: HSV-1 F: protein variant, $F(3,24)=41.951$, $p<0.0001$; dose, $F(2,24)=8.625$, $p=0.0015$. HSV-2: protein variant, $F(3,24)=7.618$, $p=0.000954$; dose, $F(2,24)=13.153$, $p=0.000139$. GST and MxB protein expression was controlled by immunoblot analysis using pooled samples for each condition. GAPDH served as loading control. **b** HeLa cells were transiently transfected with pIS1-Eef25UTR-renilla constitutive reporter plasmid together with increasing amounts of expression plasmids encoding the indicated proteins. Constitutive reporter activity was determined by luciferase assay at 32 h post transfection and is presented relative to the reporter only control. Bars indicate mean+s.d., $n=3$ biological replicates. Additive ANOVA model with protein variant and dose as explanatory variables: protein variant: $F(4,41)=2.996$, $p=0.0294$; dose: $F(2,41)=0.05$, $p=0.9513$. GST and MxB protein expression was controlled by immunoblot analysis using pooled samples for each condition. GAPDH served as loading control. **c** HeLa cells were transiently transfected with pGL-T9G reporter plasmid. At 24 h post transfection, cells were mock-infected or infected with HSV-1 strain MacIntyre (MOI=0.5) in presence of increasing concentrations of the HSV-1 DNA polymerase inhibitor phosphonoacetic acid (PAA). Virus infection efficiency was determined by luciferase assay at 24 h post infection and is presented relative to the condition without PAA. Bars indicate mean+s.d., $n=3$ biological replicates. One-way ANOVA modelling PAA concentration as explanatory variable: $F(5,12)=115.2$, $p<0.0001$. HSV-1 immediate-early protein VP22 and late protein VP16 expression was controlled by immunoblot analysis using pooled samples for each condition. GAPDH served as loading control. **d** HeLa cells were transiently transfected with pGL-T9G reporter plasmid. At 24 h post transfection, cells were mock-infected or infected with HSV-1 strain MacIntyre (MOI=0.5). Virus infection efficiency was determined by luciferase assay at the indicated time points post infection and is presented relative to mock-infected cells. Bars indicate mean+s.d., $n=6$ biological replicates. One-way ANOVA modelling time of infection as explanatory variable: $F(4,25)=226.9$, $p<0.0001$. **e** HeLa cells were transfected as in **d** but then mock-infected or infected HSV-1 strain MacIntyre at increasing MOI. Virus infection efficiency was determined by luciferase assay at 32 h post infection and is presented relative to mock-infected cells. Bars indicate mean+s.d., $n=3$ biological replicates. One-way ANOVA modelling MOI as explanatory variable: $F(6,14)=101.8$, $p<0.0001$. Multiple comparisons: NS $p\geq0.05$; *, $p<0.05$; ** $p<0.01$; *** $p<0.001$; and **** $p<0.0001$.

Supplementary Fig. 7

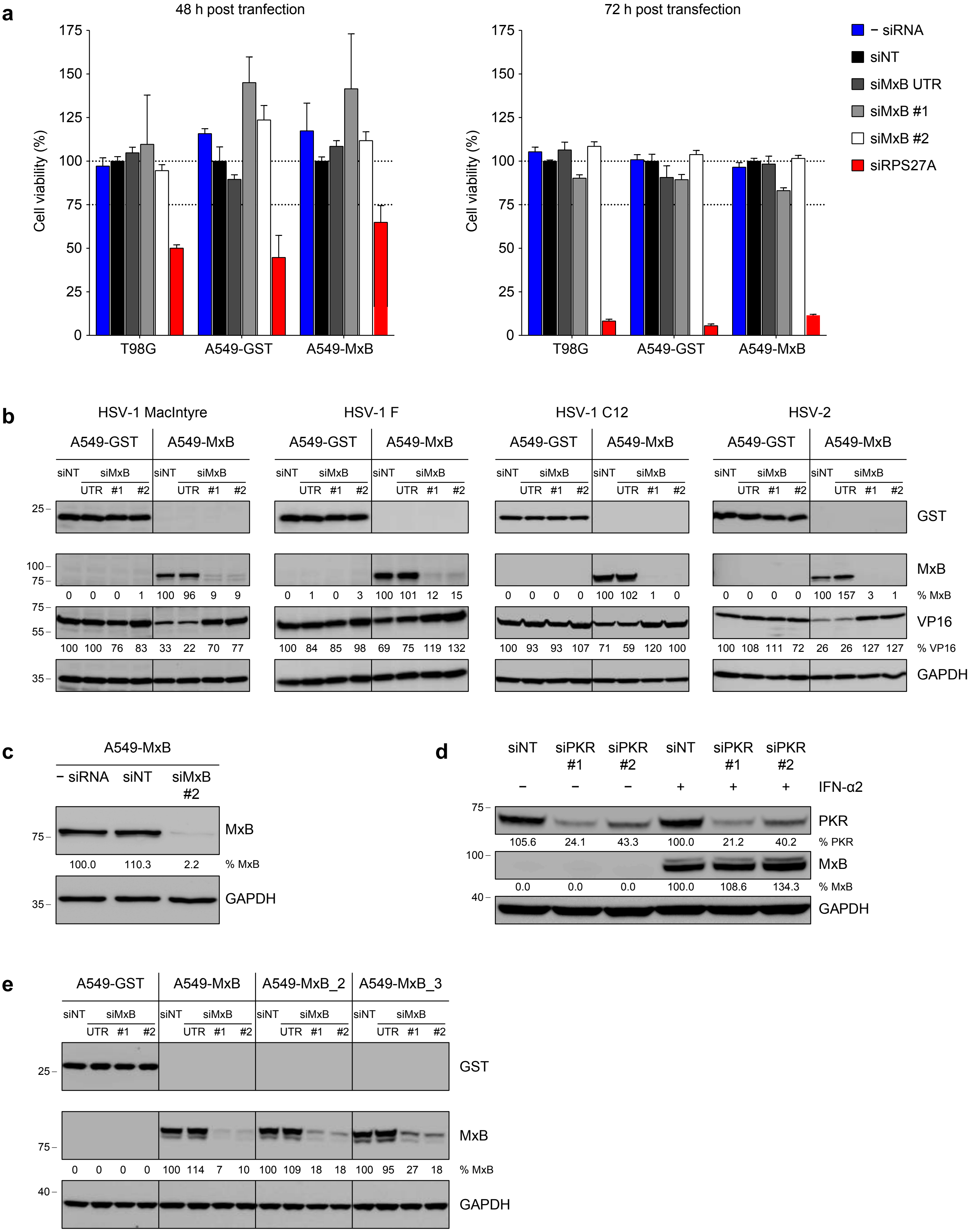

Supplementary Fig. 7 Control experiments to assess cell viability, *MX2* and *PKR* silencing efficiency, and HSV protein expression. **a** T98G, A549-GST, and A549-MxB cells were mock-transfected or transfected with the indicated siRNAs. Cell viability was assessed at 48 h (left panel) and 72 h (right panel) post transfection by measuring the levels of intracellular ATP using the CellTiter-Glo® luminescent Cell Viability Assay (Promega). As a positive control, siRPS27A was used to target ribosomal protein S27A, thus leading to cell death. Dotted lines indicate 75% and 100% cell viability relative to siNT-transfected cells. Bars indicate mean+s.d., *n*=3 biological replicates. **b** Refers to Fig. 2f-i: A549-GST and A549-MxB cells were transfected with the indicated siRNAs. At 48 h post transfection, cells were infected with HSV-1 strain MacIntyre or F (MOI=0.05) for 24 h, recombinant strain C12 (MOI=0.5) for 24 h, or HSV-2 strain G (MOI=0.05) for 48 h. Cells were lysed and pooled for immunoblot analysis of GST and MxB protein expression, *MX2* silencing efficiency, and HSV late protein VP16 expression. GAPDH served as loading control. **c** Refers to Fig. 6: A549-MxB cells were mock-transfected or transfected with the indicated siRNAs. At 60 h post transfection, cells were lysed for immunoblot analysis of *MX2* silencing efficiency. GAPDH served as loading control. **d** Refers to Supplementary Fig. 1c: T98G cells were transfected with the indicated siRNAs. At 30 h post transfection, cells were mock-stimulated or stimulated with human IFN-α2 (500 IU ml^{-1}) for 18 h. At 80 h post transfection, cells were lysed and pooled for immunoblot analysis of MxB protein expression and *PKR* silencing efficiency. GAPDH served as loading control. **e** Refers to Supplementary Fig. 2a: A549-GST and A549-MxB cells plus two additional independent high-expression MxB clones (A549-MxB_2 & A549-MxB_3) were transfected with the indicated siRNAs. At 72 h post transfection, cells were lysed and pooled for immunoblot analysis of GST and MxB protein expression and *MX2* silencing efficiency. GAPDH served as loading control.

Supplementary Fig. 8

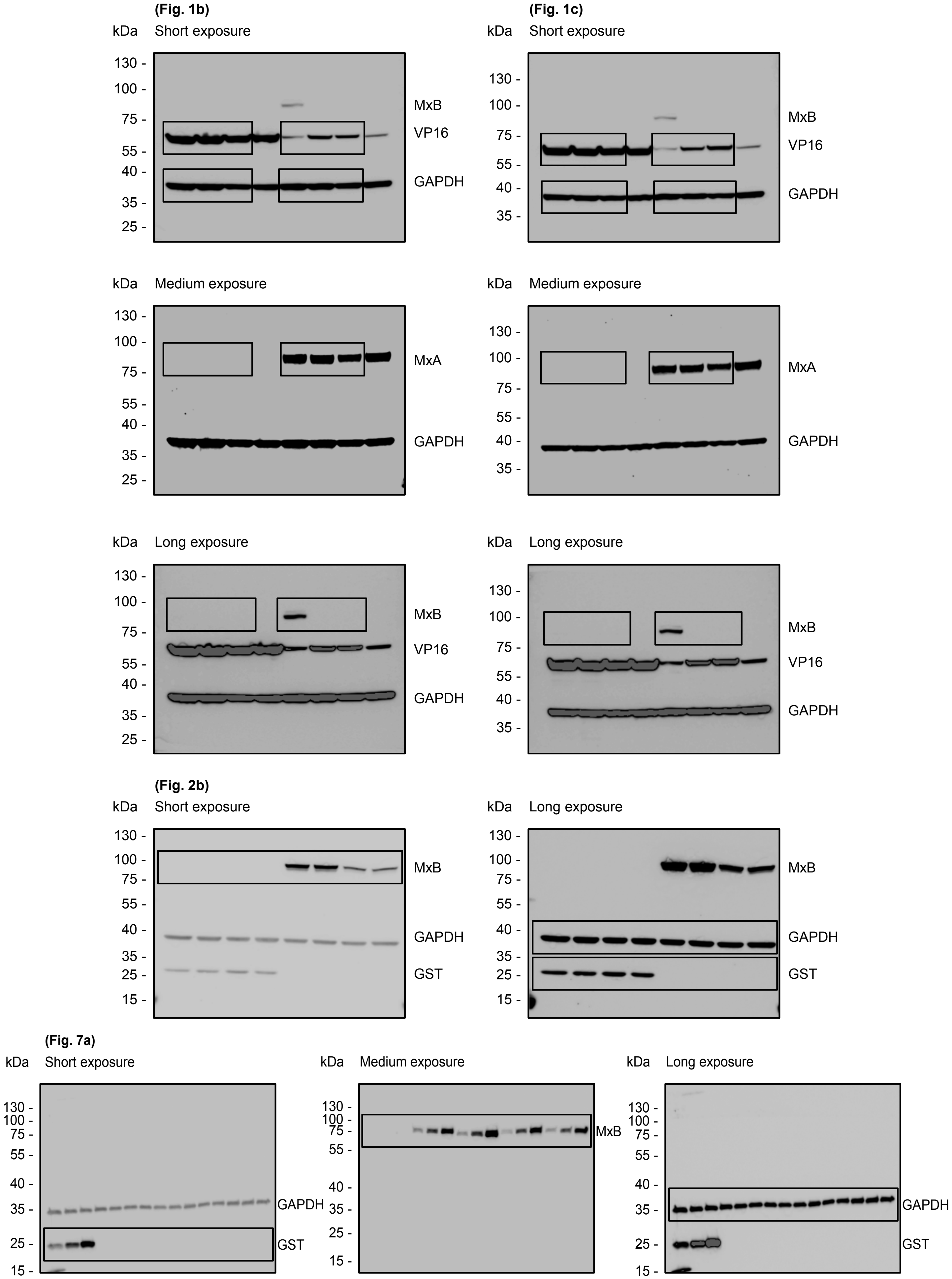

Supplementary Fig. 8 Uncropped scans of immunoblots shown in Fig. 1a, b, Fig. 2b, and Fig. 7a.

Supplementary Fig. 9

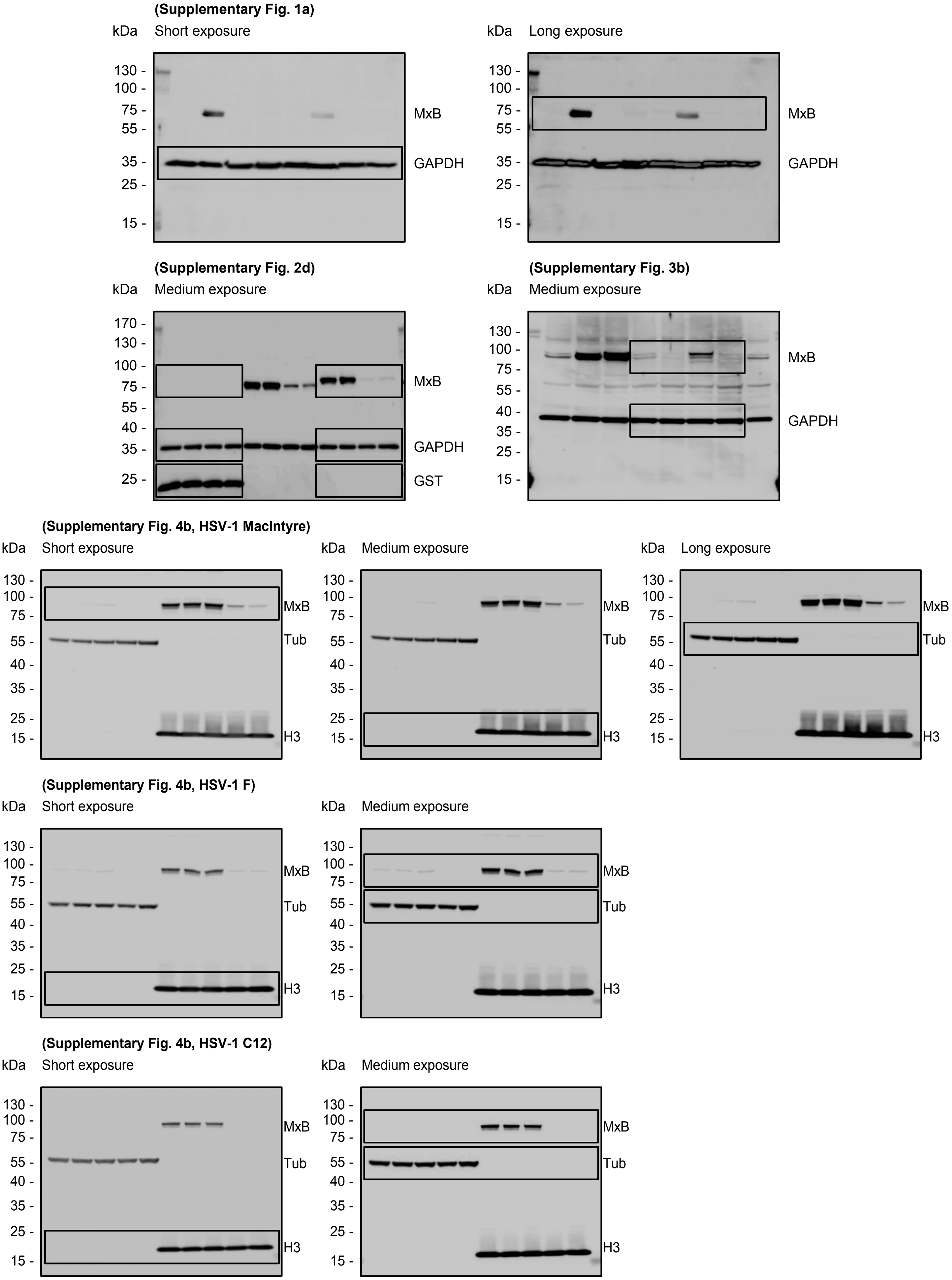

Supplementary Fig. 9 Uncropped scans of immunoblots shown in Supplementary Fig. 1a, Supplementary Fig. 2d, Supplementary Fig. 3b, and Supplementary Fig. 4b.

Supplementary Fig. 10

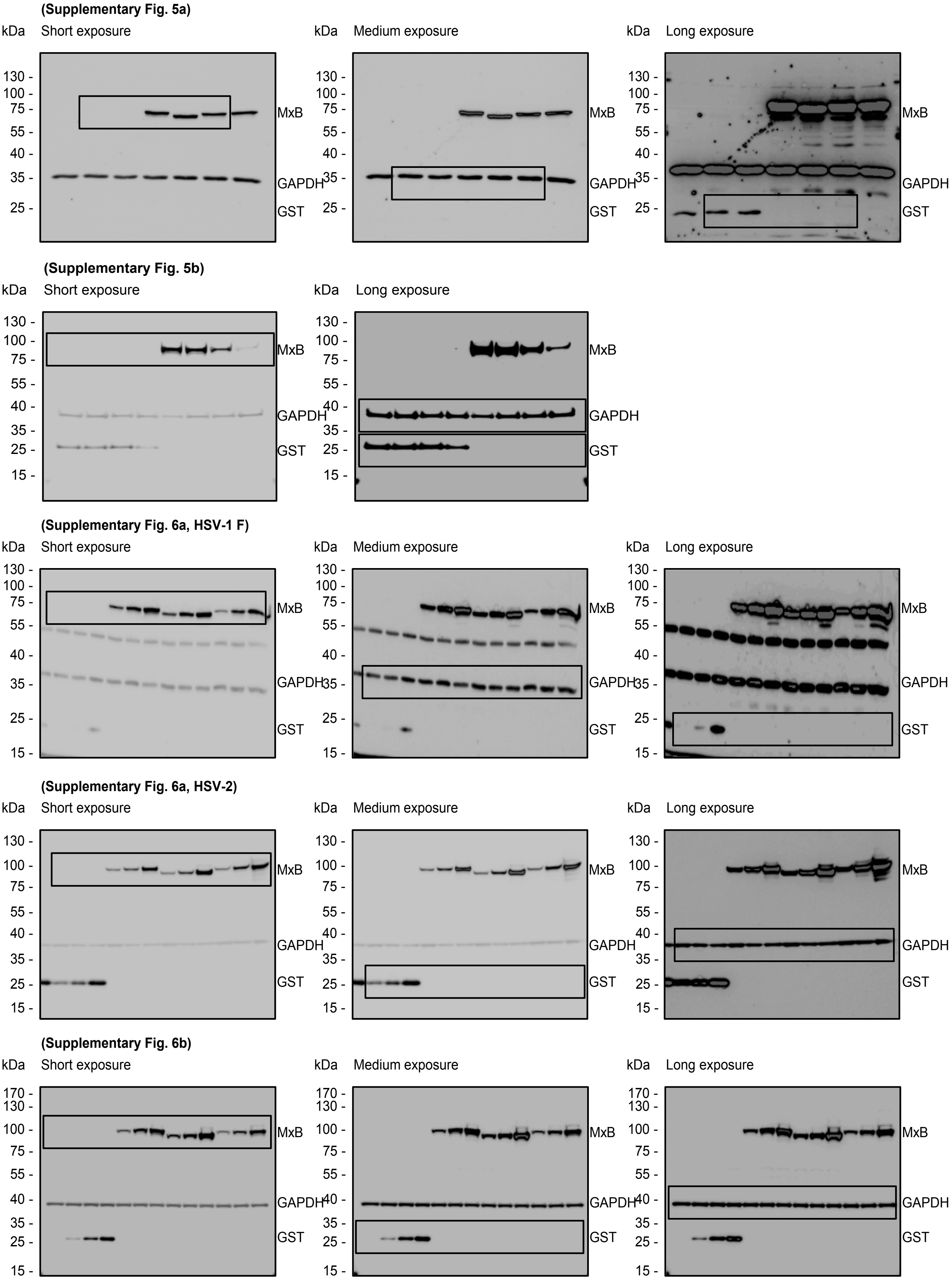

Supplementary Fig. 10 Uncropped scans of immunoblots shown in Supplementary Fig. 5a, Supplementary Fig. 5b, Supplementary Fig. 6a, and Supplementary Fig. 6b.

Supplementary Fig. 11

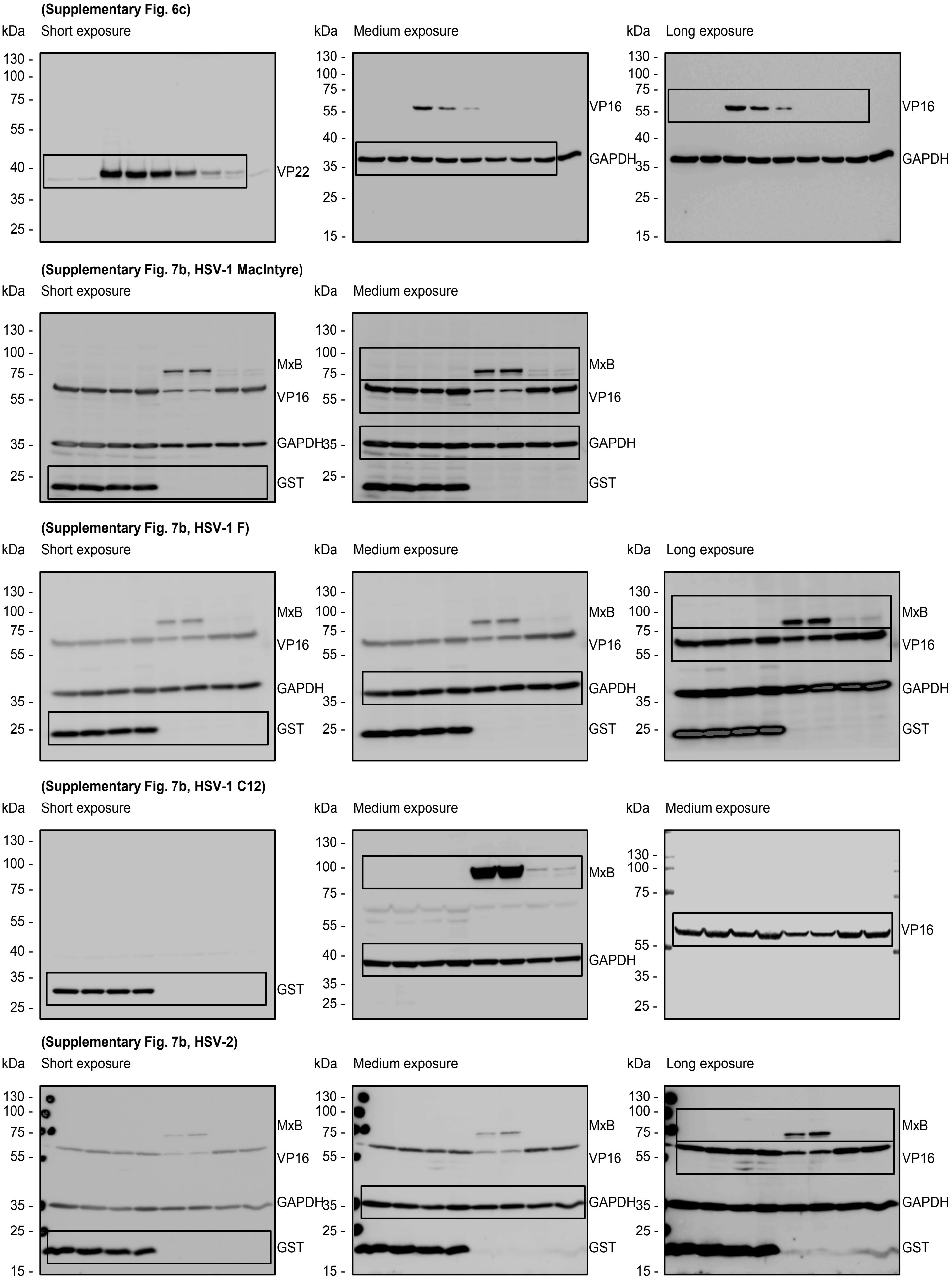

Supplementary Fig. 11 Uncropped scans of immunoblots shown in Supplementary Fig. 6c and Supplementary Fig. 7b.

Supplementary Fig. 12

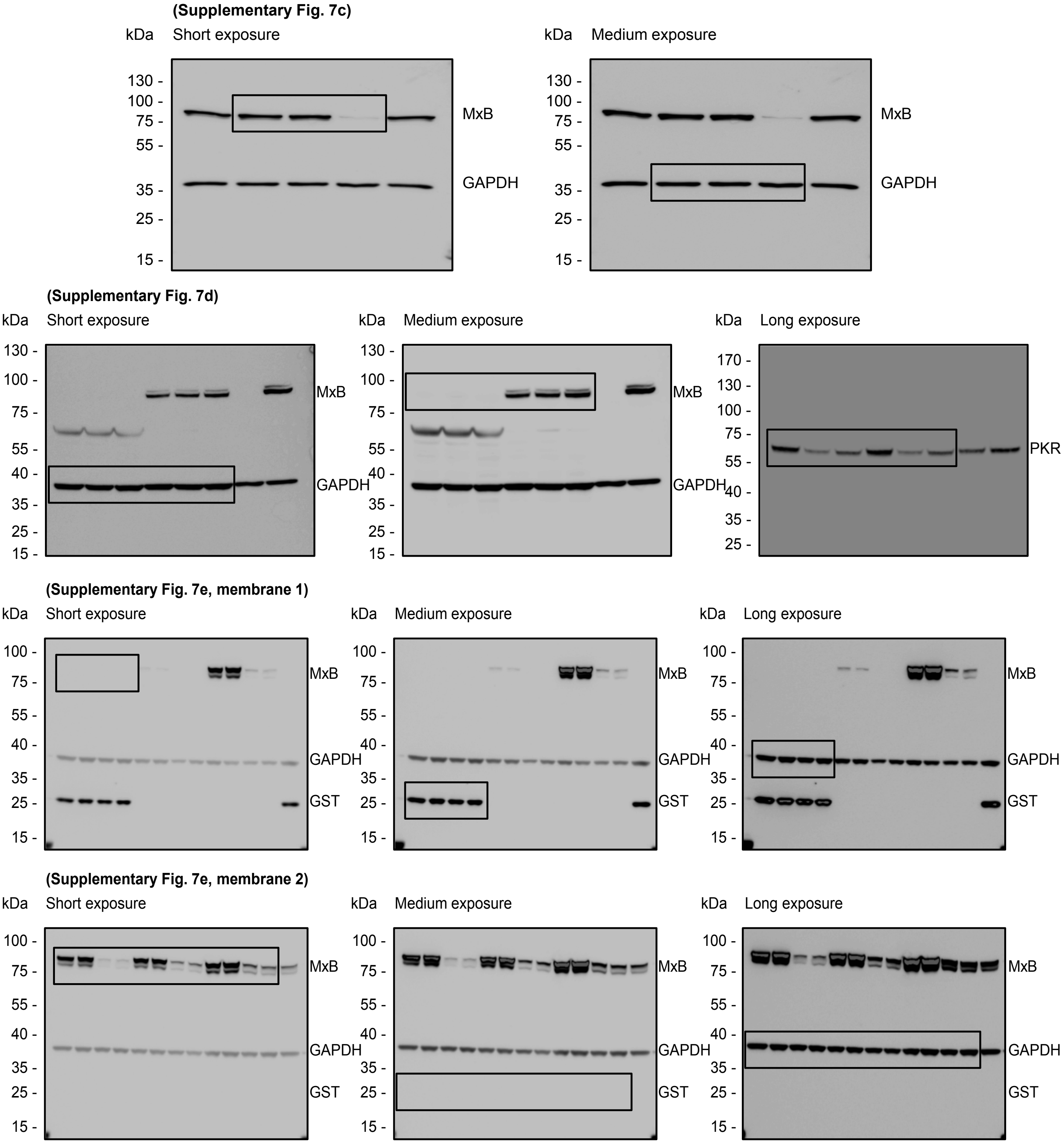

Supplementary Fig. 12 Uncropped scans of immunoblots shown in Supplementary Fig. 7c, Supplementary Fig. 7d, and Supplementary Fig. 7e.
